# Community stakeholders’ perspectives on the role of occupational therapy in primary healthcare: Implications for practice

**DOI:** 10.4102/ajod.v6i0.255

**Published:** 2017-02-28

**Authors:** Deshini Naidoo, Jacqueline Van Wyk, Robin Joubert

**Affiliations:** 1 School of Health Sciences, University of KwaZulu-Natal, South Africa; 2 School of Clinical Medicine, University of KwaZulu-Natal, South Africa

## Abstract

**Background:**

Primary healthcare (PHC) is central to increased access and transformation in South African healthcare. There is limited literature about services required by occupational therapists in PHC. Despite policy being in place, the implementation of services at grassroots level does not always occur adequately.

**Objectives:**

This study aimed at gaining an understanding of the challenges of being disabled and the services required by occupational therapists (OTs) in rural communities in order to better inform the occupational therapy (OT) training curriculum.

**Method:**

An exploratory, descriptive qualitative design was implemented using purposive sampling to recruit 23 community healthcare workers from the uGu district. Snowball sampling was used to recruit 37 members of the uGu community, which included people with disability (PWD) and caregivers of PWDs. Audio-recorded focus groups and semi-structured interviews were used to collect data, which were thematically analysed. Ethical approval was obtained from the Biomedical and Research Ethics Committee of the University of KwaZulu-Natal (BE248/14).

**Results:**

Two main themes emerged namely: firstly, the challenges faced by the disabled community and secondly appropriate opportunities for intervention in PHC. A snapshot of the social and physical inaccessibility challenges experienced by the community was created. Challenges included physical and sexual abuse, discrimination and marginalisation. Community-based rehabilitation and ideas for health promotion and prevention were identified as possible strategies for OT intervention.

**Conclusion:**

The understanding of the intervention required by OT in PHC was enhanced through obtaining the views of various stakeholders’ on the role. This study highlighted the gaps in community-based services that OTs should offer in this context.

## Introduction

The right to equitable health services for people with disabilities (PWDs) is supported by South Africa’s (SA) commitment to the UN convention on the rights of PWDs and the SA constitution (McIntyre & Atatguba 2012; Sherry 2014). The inequitable access to health services for PWDs is well documented in recent research (Duncan et al. 2012; Harris et al. 2011; Van Rooy et al. 2012). But despite the country’s commitment to PWDs at a policy level, the implementation of these policies is still problematic. Barriers such as the lack of transport, extended waiting periods, negative attitudes of the public and lack of knowledge among healthcare workers have been reported (Maart et al. 2007; McIntyre 2012; Mji et al. 2009; Moodley & Ross 2015; Scheffler, Visagie & Schneider 2015; Visagie & Schneider 2014).

Research by Duncan and Watson (2009) has demonstrated the strong links between disability, poverty and physical contextual challenges which impact on PWDs. These factors are further compounded through PWDs exclusion from social and economic opportunities and their increased risk of contracting non-communicable diseases (Coovadia et al. 2009; Duncan et al. 2012). PWDs are also more vulnerable to physical, sexual and psychological abuse, and they are often easily exploited (Neille & Penn 2015a, 2015b). Social marginalisation, material deprivation added to limited access to education and infrastructural challenges such as poor roads, costly transport, inaccessible terrains and poor access to health services contribute towards feelings of powerlessness, vulnerability and lack of voice of PWDs (Duncan et al. 2012; Jelsma 2007; Kahonde, Mlenzana & Rhoda 2010; Lorenzo 2012; Neille & Penn 2015a).

After 1994, a geographically based district health system was introduced to provide public healthcare. Service delivery in the SA health system is offered at tertiary, secondary, district and primary levels of care. Efforts to promote the principle of ‘Health for all’ have seen the implementation of the National Health Insurance (NHI) by the Department of Health (DoH 2011). The objectives of the NHI include improved access to quality health services, especially for vulnerable populations such as PWDs, social redress in the health system, procuring services on behalf of the population and the strengthening and delivery of more efficient public services (DoH 2015). This led to a DoH strategy that strongly advocates for the provision of rehabilitation services in community settings and an adherence to a primary healthcare (PHC) approach. This strategy included all therapists assigned to district- and secondary-level hospitals, in order to offer more appropriate rehabilitation services (DoH 2015).

This aimed at improving access to healthcare for people in communities and made a shift from primarily offering a curative and medical focus to one where the emphasis was on prevention of disease and disability, promotion of health and well-being and rehabilitation for those affected by disability (DoH 2011). DoH policies recognised the need for rehabilitation, intersectoral collaboration and programmes to promote health and facilitate social inclusion of PWDs. The National Rehabilitation Policy (NRP) views ‘rehabilitation’ as an important component of PHC (DoH 2000). Both the Integrated National Disability Strategy (INDS) and the NRP are based on a social model of disability and advocate for rehabilitation services to facilitate empowerment, collaboration within sectors and the community, social inclusion and to encourage PWDs to participate in daily activities to allow for integration in all spheres of life including social, economic, education and recreation (DoH 2000; South African Government 1997).

The WHO (2010) and the NRP (2000) identified community-based rehabilitation (CBR) as a strategy to improve access to rehabilitation and to foster community development, equality, poverty reduction and social inclusion of PWDs. Both the INDS and the NRP recognise the role of rehabilitation in improving the PWDs’ physical, psychosocial and vocational well-being and its role in improving their overall quality of life (DoH 2000; South African Government 1997).

Despite the existence of policies with good intent, there is generally poor implementation and a lack of translation of such policies/frameworks into practice (Visagie & Schneider 2014). Current therapy services at a PHC or CHC level tend to provide individual facility-based rehabilitation sessions focused on treating acute conditions such as stroke and rheumatoid arthritis in twice monthly or monthly sessions (Scheffler et al. 2015). Furthermore, there appears to still be an entrenched adherence to the medical model with ‘lip service’ being paid to CBR in SA, which manifests itself in OTs resorting to the familiar hospital care approach even in settings requiring a different strategy. This suggests support for the principle but not necessarily a readiness to abandon the comfort of institution-based rehabilitation services. Wilding (2011) found that OTs unconsciously conform to hospital-based protocol, which hinders occupation-based practice. Likewise, Méthot (2004) found that the dominant medical epistemology hinders occupational therapists being able to apply health promotion and disease prevention strategies. A ‘one size fits all’ approach, which borrows heavily from the Western, and mostly Eurocentric, rehabilitation approaches and is applied in some developing country’s approaches to rehabilitation such as Asian and South American countries (Fransen & Van Riel 2005; Thompson et al. 2010; Van Bruggen 2010), does not always apply to the SA context. These approaches do not accommodate the unique, idiosyncratic concerns that are relevant to the Southern African context; for example, the effects of environmental factors, such as the rural terrain and inadequate resources, on PWDs ability to perform basic daily activities, as well as taking into consideration the unique African cultural beliefs about disability and health.

The main goal of occupational therapy (OT) is to promote health and well-being through enabling individuals with- or at risk of- disability to engage in life-sustaining and health-promoting occupations. It achieves this goal through the facilitation of daily activities that promote physical and psychological well-being such as self-care and vocational tasks (World Federation of Occupational Therapy [WFOT] 2010). OT education and practice for service delivery at a PHC level has thus shifted its focus from education with a narrow technical focus to encompass a more comprehensive occupational justice framework, where members of a community have equal opportunities for meaningful and satisfying participation in their daily activities. The main premise of this framework considers how physical, social, political and emotional barriers impact on the individual’s ability to access community services for participation. Being denied opportunities to participate in meaningful daily life activities results in occupational restriction and concomitant marginalisation of the PWD, which may result in additional illnesses at a psychological and physical level (Whiteford 2004; Wilcock & Hocking 2015). Visagie and Schneider (2014) reported that current interventions at a PHC level were not client-centred and there was not proper collaboration with PWDs during treatment planning. Furthermore, language barriers, a failure to seek the goals of users, adherence to technical, curative interventions with little consideration of the determinants of ill health and insufficient explanations to PWDs about possible interventions often limits the effectiveness of intervention given at PHC level.

To be effective in delivering within a PHC service, occupational therapists (OTs) have to re-evaluate the models used for practice in the community. They need to refocus their efforts on enabling greater participation for PWDs in daily activities despite their experiences of physical, socio-political and psychological barriers. Additionally, OTs need to focus on prevention of secondary and tertiary complications such as development of pressure ulcers and contractures and promotion of health and well-being (e.g. improvement of quality of life through social inclusion). OTs also have to demystify unfounded beliefs or stereotypes and address the community’s reactions to PWDs and improve their living conditions, thereby advocating for their basic rights (Lorenzo 2012; Rule 2008; Watson & Swartz 2004).

The changing face of PHC delivery in SA has necessitated the review of older principles and approaches such as Alma Ata, to accommodate challenges unique to the country (Scheffler et al. 2015; Visagie & Schneider 2014). For example, health professionals such as OTs need to identify community-specific determinants of the burden of disease. OTs need to assist the community with development and implementation of health promotion programmes that address the identified community-specific determinants and health behaviours related to human occupation with due consideration of the dominant culture within that community.

OT training is often accused of having an overloaded curriculum that does not allow students adequate time to assimilate much of their knowledge. The unburdening of the curriculum requires a refinement of existing modules and essential components such as PHC to fit into existing time constraints. In addition to this, the rapidly changing health delivery scenario, since 1994, and the need to ‘keep up’ with these changes, has often lead to the dilution of aspects of PHC and even to losses of essential concepts, while OT training centres stagger under the increasing demands to stay abreast and be relevant in their training. Guidelines for training and education of OTs in SA include adherence to a comprehensive PHC approach and its core principles of CBR, the rights-based and integrated multidisciplinary approaches (OTASA 2015). These policies, when applied, change what OTs are expected to be doing when working in the community. For example, OTs are expected to accept non-traditional roles in new complex environments such as being the project facilitator in a secondary prevention initiative aimed at stimulating children with disabilities rather than offering direct therapy (OTASA 2015). Also, OTs are expected to initiate and participate in intersectoral collaborations (e.g. needing to negotiate with local counsellor or municipal transport services that offers affordable transport for PWDs to PHC clinics) (OTASA 2015).

The policies promote a re-conceptualisation of the OT role in PHC to include a greater advocacy for PWDs, promoting their social reintegration in activities and collaboration with members of the community in project development. Anecdotally, many community service therapists can only visit a clinic once every 6 weeks because of the large number of clinics to be visited and the shortage of OTs working in the community. These community service OTs are currently only delivering rehabilitation services and conducting home visits while assigned to PHC clinics, which is only partially aligned to the reconceptualised OT community service.

OT graduates from the University of KwaZulu-Natal (UKZN) predominantly spend their first year of work in the KwaZulu-Natal (KZN) province while employed in the DoH. The University has a memorandum of understanding to train graduates for service in decentralised and rural PHC settings as part of the KZN DoH’ s strategic plan to improve training of health graduates and provide greater access to health services in rural areas(KwaZulu-Natal DoH 2014). This study targeted the district of uGu, in KZN, because it represents a typical district level site where UKZN OT graduates provide community service. Hospitals in the uGu district are also being considered for a decentralised undergraduate training platform, that is, satellite service-learning sites in rural or underserved areas. This paper formed part of a larger study that was aimed at creating contextual evidence to inform the graduate competencies needed for effective OT service delivery at a PHC level. The purpose of this particular paper is to describe the challenges experienced by community stakeholders, that is, uGu community members with disability, their caregivers and the community healthcare workers (CHWs) who work with PWDs in order to better understand what community services are required of OT.

This paper argues that there are still gaps in the service OTs can and should be offering in this particular context and that OT practice needs to change to deliver a more relevant service.

## Research methods and design

### Study setting

The study was conducted in the province of KZN because the occupational therapy programme of the UKZN requires graduates to provide mandatory year of community service to peri-urban and rural hospitals in this region. All SA-trained health professionals have to provide a mandatory year of community service in a public health facility, generally at a district level (South African Government 2002). This policy was implemented to increase the provision of health services to communities, mostly at a district level where disadvantage is high and in rural and peri-urban areas in SA. While there are usually a doctor and other rehabilitation professionals, the OTs may sometimes be the sole therapists in the area and often have to work under the supervision of another health profession, for example, the medical manager.

The uGu district has been classified as a designated rural node for PHC delivery. Designated rural nodes refers to rural areas where population is predominantly rural, there are low levels of income (R500 to R1200 per month), extreme poverty and high dependency rates (858 youths below 14 years per 1000 people in age category 15–65). Additionally, there is fragmented service delivery by different governmental spheres, high unemployment rates, low levels of formal education and high dependency on social welfare grants (Harmse 2010; Pillay 2007). The uGu district and the hospitals and clinics within it are being developed as a decentralised training site for undergraduate students. Therefore, this study will assist in informing the graduate competencies these community service OTs need to acquire in order to work in settings such as this.

UGu Health District is located in the lower South Coast region of the province of KZN and is geographically 16% urban and 76% rural areas. It has a population of approximately 722 484 people with 60% of the population aged 15–64 while 33% of the population is younger than 15 years old (Local Government Handbook 2016). uGu has an unemployment rate of 35% with an average income per capita of between R800 and R2953 per month. Of the households, 24% have access to piped water inside their homes and only 18% have access to flushing toilets (Local Government Handbook 2016). Health services are provided using a Primary Health Care approach through the district health system, which involves all levels of care (KZN DoH 2016). The geographically based district health system offers services at quandary, tertiary, secondary, district and primary levels of care (Couper & Hugo 2005). The uGu Health District has two district and one regional hospital staffed by both permanent and community service occupational therapists (KZN DoH 2016).

### Design

A descriptive qualitative study was used to explore the perceptions of community members and CHWs from the uGu district, on their experiences of coping with disability and the services required from OTs to assist them. An isiZulu-speaking research assistant who had experience in conducting research for the UKZN rural health centre and was familiar with the community and the clinics in the northern and southern uGu district assisted in gathering data. The research assistant recruited participants for the study, facilitated the audio-recorded semi-structured interviews and focus groups and collected the cameras with the pictures that the community members took. Her knowledge of the community and use of isiZulu to collect the data was aimed at maximising trust and ensuring clarity of understanding. The principal researcher was an observer in the focus groups and visited some homes of the participants with the research assistant acting as interpreter in these instances. This was done to allow the principal researcher to immerse herself in the study setting, in order to better understand the context and the challenges experienced by the PWDs. Data collection was done over a 3-month period.

### Participants, sampling and recruitment strategies

A purposive sampling strategy was used to recruit CHWs who worked with PWDs and who either worked for or visited the eight PHC clinics served by the community service occupational therapists. The research assistant explained the purpose of the study to CHWs at each PHC clinic with a view to recruit them for participation on the study. Twenty-three CHWs were selected and consented to participate in the study.

Snowball sampling was used to recruit community members with disability and their caregivers from the community. The participants were deemed suitable for inclusion if they were aged 19–70, lived in uGu north and south and attended one of the clinics served by the occupational therapists working in the district. The CHWs suggested potential participants from the community for the study who were invited to a meeting during which the research assistant explained the purpose of the study and the requirements of the study was explained. From the five recruitment meetings, 37 members of the community (PWDs and their caregivers) aged 19–70 were finally selected and consented to participate. Apart from participating in interviews, the community participants were also given cameras to document challenges they experienced and the ethics around taking photographs were explained. By taking photographs participants were able to represent their views about health and occupations that were relevant to them (Hergenrather et al. 2009). The photographs were used for discussion in the focus groups.

### Procedure

Community participants met at local venues for the five audio-recorded focus groups of approximately an hour, which were conducted in isiZulu by the research assistant with the principal researcher attending as an observer. Similarly, four focus groups for CHW were conducted (see Table 1). In the community member focus groups, the research assistant used the photographs taken by the participants to commence the discussion around the challenges they experienced, the occupations they did and had difficulty with and their ideas of the services OTs could offer. Most of the participants had seen a therapist at least once at a PHC clinic or at the hospital. The assistant asked participants if they knew what an occupational therapist was prior to starting the focus group, and if anyone did not know, she provided an explanation of the profession.

**TABLE 1:** Participant description and data collection methods.

Participants	Data collection method	Procedure	Core questions
**Community /disabled members** (*n* = 37) Gender: Female (*n* = 23) Male (*n* = 4) Ages: 19–30 years (*n* = 6) 31–40 years (*n* = 11) 41–60 years (*n* = 16) 61–70 years (*n* = 4)	Five **Focus Groups** with 5–6 members of community in uGu District.	There were three meetings conducted by the research assistant for each of the focus groups. To discuss the purpose of the research, obtain consent and explain about taking photographs and ethics around this To collect the cameras to develop the pictures To facilitate the focus group which lasted for 45–60 minutes in duration and were conducted in isiZulu The principal researcher was an observer in the focus group	Can you explain the challenges you experienced? What services do you think that OTs should offer in the community?
	Seven **Semi-structured interviews** were conducted with members of the community in uGu.	The research assistant conducted the interviews lasting for 30–45 minutes in isiZulu.The principal researcher conducted five participant observations by visiting their homes with the isiZulu-speaking research assistants and an interpreter.	To interrogate the themes that emerged from the focus groups (above).
**Community healthcare worker (CHW)** (*n* = 23) Gender: Female (*n* = 21) Male (*n* = 2)	Four **Focus groups** with CHWs.One conducted in English by the researcher.	The research assistant conducted the focus groups interviews lasting for 45–60 minutes in isiZulu with 5 CHWs in each group.The principal researcher observed two focus groups.	What challenges do PWDs experience? What services do you think that OTs should offer in the community?
Ages: 31–40 (*n* = 13) 41–60 (*n* = 10)	Two **Semi-structured interviews** were conducted with CHW.	The semi-structured interviews were 30–45 minutes in duration. One was conducted in isiZulu by the research assistant and one in English by the principal researcher.	To interrogate and ratify information from the themes that emerged from the focus group.

The community PWDs and their caregivers were grouped according to age, for example, 19–40, 41–70, for each focus group in order to explore perceptions of both younger and older participants. It also allowed the younger members to participate more openly without being inhibited by their elders during the focus groups, this conforming to the isiZulu cultural norms of respect. There were a maximum of five participants’ in both the focus groups of the community members and the CHWs. The questions posed to both sources centred on their perceptions of health, the daily activities that they had difficulty performing and the services they felt they required from OTs.

Additional community participants were selected for the semi-structured interviews, which were aimed at elaborating on the themes that emerged from the focus group. Eight semi-structured interviews took place in participants’ homes. These interviews were conducted for 45–60 minutes and were conducted by the research assistant in isiZulu. The audio-recorded data were transcribed, translated into English and verified.

The observations in community participants’ homes were conducted concurrently with the semi-structured interviews during which the researcher spent time observing the participants’ performing their daily activities. These observations helped to establish how the person with a disability dealt with the various challenges within their environment and how others in the community related to them. The information gained re-enforced the information collected from the photographs and the focus groups, which assisted in triangulation of the data and robustness of the data.

### Data analysis

Data were analysed using inductive thematic analysis (Miles, Huberman & Saldana 2014; Creswell 2013). The steps of data analysis included familiarising the researcher with the data, writing notes, developing codes and noting patterns and themes (Creswell 2013). Each of the 18 transcripts was analysed separately. The identified categories across all transcripts (18 transcripts) were then grouped according to recurring patterns into themes by the principal researcher. A third round of analysis yielded two main themes; namely, the challenges experienced with engaging in occupations and how OT intervention and services could address these challenges. The principal researcher met with a co-researcher who had not been part of the data collection in order to discuss the nature of the findings and attain consensus with regard to the emerging themes.

Themes and subthemes that emerged provided insight into the challenges faced by PWDs in rural and semi-rural areas. Verbatim quotes were used to maintain the voices of the participants.

## Ethical considerations

Ethical clearance was obtained from the Biomedical Research Ethics committee of the UKNZ (BE248/14). Gatekeeper permission was sought from the uGu Health District, hospitals to which the CHWs were assigned and the KZN provincial ethics office prior to commencement of the study. Additionally, permission was sought from the local leader in the areas where the focus groups were held in uGu north and south to gain permission for community entry and to access people in the community for the study. Participation in the study was voluntary. The consent form was read and explained to illiterate participants who signed consent using an ‘X’. All data were treated as confidential and the participants were given pseudonyms during analysis and write-up. Written permission was sought and received from participants for the use of the photographs and pictures used in this publication. All the interviews were conducted at an accessible venue and participants were reimbursed for travel costs from a Medical Education Partnership Initiative grant. Refreshments were also served to all participants.

### Trustworthiness

Confirmability, triangulation and trustworthiness were ensured through an audit trail, by collection of data from multiple sources and through the observations conducted by the principal researcher. Key points from the focus group or interviews were repeated at the end of the interview to allow for verification through member checking to ensure an accurate reflection of the discussion. The isiZulu data were transcribed in isiZulu, translated into English and then back-translated to ensure veracity of the data. The researcher kept a reflexive journal during the data collection and data analysis process to minimise bias. The reflexive journal entailed the primary researcher expressing her assumptions and experience though a reflexive statement prior to beginning the research and continued as her writing analytical memos during the data gathering and analysis. This process of documenting decisions made contributed towards confirmability of the data.

## Findings and discussion

### Demographic characteristics of the participants

The participants represented PWDs, caregivers and CHWs from peri-urban and rural districts of uGu in KZN. The ages of participants ranged between 19 and 70 with the majority ranging from 40 to 60 years. The disabled community participants had predominantly physical disabilities with the exception of one person who was partially sighted and two people who had mental health disorders. The main source of income for participants was a disability grant and the average income per household ranged between R1900 and R2000 per month. Judging from their income and living conditions, this group could mostly be classified as living in poverty as defined by the United Nations Economic and Social Council (1998):

a denial of choices and opportunities, a violation of human dignity, lacking in the basic capacity to participate effectively in society and not having enough to feed and clothe a family. It also implies difficulty in accessing educational, social and health structures such as schools or clinics. … It means insecurity, powerlessness and exclusion of individuals, households and communities. It means susceptibility to violence, and it often implies living on marginal or fragile environments. (p. 1)

The feedback from this group of inter-related community stakeholders provided a fair account of the challenges that PWDs face in this community. However, because some participants had minimal contact with, or knowledge of OT, the comprehensiveness of their insight into the occupational therapist’s role and scope was limited. In spite of this, they were able to provide a snapshot of the occupational and social challenges faced by PWDs of that district, which allowed for insights into issues such as human rights, the effects of being denied the right to participate in daily activities and the role of OTs working in similar PHC settings.

Two main themes, each with respective subthemes, emerged from the interviews and focus groups discussions. These were:

- the challenges faced by PWDs and their caregivers, and
- suggestions by participants about opportunities for OT interventions to address the challenges experienced by PWDs.

*Accessibility* was a sub-theme that emerged in discussions on the challenges experienced by PWDs. Not only did accessibility relate to essential resources such as clinics and shops but also access to resources such as water and firewood, which are essential for survival in the setting. For some, these challenges were aggravated by high transport costs, the geographically difficult terrain typical of rural KZN and limited financial resources as indicated in the quote below:

‘I struggle with water so I end up hiring someone to fetch water for me.’ (Participant 26, Female PWD, Community focus group 5)

Another access challenge related to the financial and terrain constraints faced by PWDs living in such areas. These include the often undignified ways in which they are transported such as in a wheelbarrow or being carried piggyback by a caregiver, which affects the dignity of the PWD and is also extremely taxing on the health and well-being of their caregiver/family member. The following examples speak to these issues:

‘When we have to take her to the clinic we end up pushing her with a wheelbarrow.’ (Participant 14, Female caregiver of person with a physical disability, Community focus group 3)

‘The roads are not in a condition to push a wheelchair, so we [the caregivers] end up having to carry those people. Sometimes on their backs for a distance of ± 7 km.’ (Participant 12, Female CHW, Focus group 3)

**FIGURE 1:**
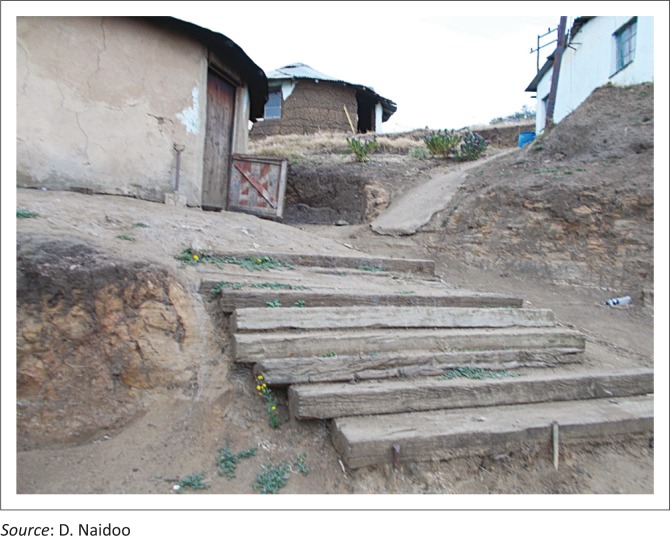
Wheel chair inaccessibility, an example of the terrain surrounding one of the homes of a person with physical disability, which limits this person’s ability to be independent in mobilising around the home.

In addition to this, general accessibility within and around the homes, mostly because of terrain and environmental obstacles seriously impact on issues of self-care such as transferring, dressing, bathing and access to toilets as demonstrated in Figure 1 to Figure 3.

**FIGURE 2:**
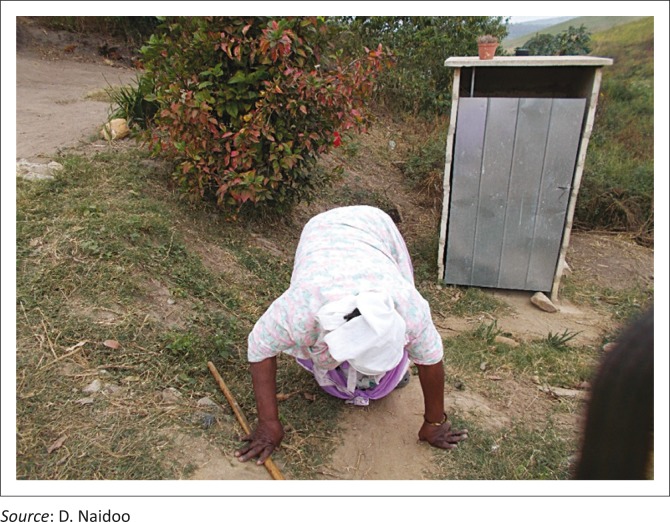
Difficulty in accessing the toilet for a person with a physical disability.

**FIGURE 3:**
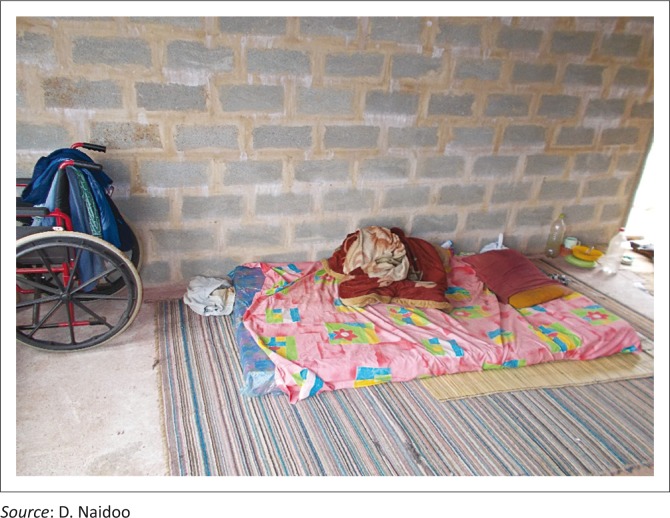
Moving oneself with a disability from a matress on the floor into a wheelchair is difficult for most PWDs and requres additional skills and strength, which is different from transferring them from a standard raised bed.

From an occupational justice perspective, these conditions impact on the ability of the PWD to access a toilet, washing and other facilities in and around the home. Participants found these challenges impacted their dignity, leaving them unable to perform those occupations essential for self-care and preparation for interpersonal interactions with others and thus also the potential to access the labour market.

The costs and demands to access transport to and from possible work places are also expensive, such that working becomes an almost impossible option. Furthermore, the authors suggest, a form of financial compensation for PWDs living in such areas, who are from low-income groups, could subsidise their transport costs or that more efficient access to public transport be made available. It is also necessary to adjust the terrain around their home environments to make these more accessible.

Occupational therapists working in PHC settings should not only be able to assess the circumstances and make realistic recommendations for alterations but they should also advocate for appropriate policy changes to support the financial needs or contextual changes to allow for greater participation of PWDs in their community and in local labour markets. For example, OTs could convince the local municipality that they have an accessible bus that picks up PWDs to transport them to the clinic.

Infrastructural challenges such as the lack of affordable transport, access to running water and electricity adds to restrictions in accessing health services and engaging in essential meaningful daily tasks for this vulnerable group of people (Duncan & Watson 2009; Townsend & Polatajko 2013; Kahonde et al. 2010). Most of the challenges of the participants were exacerbated by poverty as the participants did not have the social or economic capability to address them (Bateman 2012; Jelsma 2007; Neille & Penn 2015a; Van Rooy et al. 2012). These limitations also restrict their basic rights as stated in the SA constitution and article 19 of the UN Convention of Rights for Persons with Disabilities (South African Government 1996; United Nations Enable 2006). The restrictions also contribute to the marginalisation and isolation of PWDs and their ability to participate in meaningful roles within in their families and communities (Duncan & Watson 2009; Lorenzo et al. 2013; Shakespeare 2008; Wilcock & Hocking 2015).

The second sub-theme relating to challenges was that of *Abuse, discrimination and marginalisation of PWDs.* These acts were being perpetrated by both members from the community and families and caregivers of PWD. The community discriminated against and marginalised PWDs. Criminal elements in their communities, particularly unemployed youth who often were also substance abusers, appear to prey on the vulnerability of PWDs as illustrated in the following comment:

‘… what I [*PWD*] see here is bad because if these drug boys would come they will just push the door open because I’m using the nail as a locker [*lock*]. The other one who wears an artificial foot even made an example that if she wasn’t able to talk these boys would come and rape her without anyone knowing because she wouldn’t be able to scream for help.’ (Participant 29, Female PWD, Community semi-structured interview 1)

This particular comment highlights the lack of security and its concomitant evocation of severe stress, anxiety and fear experienced by many PWDs who become imprisoned in their homes, unable to participate safely and equally in daily community activities. These experiences concur with literature (Astbury & Walji 2014; Naidu et al. 2005)

PWDs reported abuse from family members and this was confirmed by the CHWs. This included mainly verbal abuse, neglect such as being locked-up and confined to a room, or being denied food. The latter often occurs despite the family members’ reliance on the disability grant issued to the PWD, which supports the household. The following comment demonstrates the concerns of a particular PWD participant:

‘… disabled people need some form of counselling because families gets frustrated looking after us and they end up verbally abusing us, which leads to being stressed and sometimes traumatised.; (Participant 16, Female PWD, Community focus group 3)

This highlights the importance of OTs adequately informing those they serve about their rights and how to deal with these if they are violated.

PWDs and their caregivers reported that members of the community lacked a general understanding of the PWDs condition. People of the local community often laughed at or they threatened them. The following comments demonstrate the cruelty and distress caused to PWDs by misinformed members of the community:

‘… when I hear a person laughing at me about this child, my soul is burdened with even more pain. They [*neighbours*] said ‘how is your cripple, did they call it for grant renewal’. It hurt me a lot.’ (Participant 27, Female caregiver of child with physical disability, Community focus group 5)

‘I find that people fail to understand or don’t want to understand him [*disabled child*] and that the other kids beat him.’ (Participant 19, Female Caregiver of a child with a psychical disability, Community focus group 4)

‘Even within my family there was talk that I was faking my blindness. He [*husband*] never saw the problem I was facing until the documents came back saying I was disabled.’ (Participant 12, Female person with a visual disability, Community focus group 3)

Some PWDs found it difficult to adjust to their disabled conditions and felt guilty for being a burden to their families. They felt burdensome as a member of their family assumes a caregiver role, which meant that person may have to sacrifice their schooling or employment opportunities. This concern was confirmed by caregivers who noted their own potential, personal engagements and quality of life declined when they assumed a caregiver role. CHWs added that PWDs often became socially isolated from the rest of their communities and that they required opportunities to socialise.

‘My daughter had to give up going to school to take care of me. She used to cry every day in the beginning, it’s hard for her and me.’ (Participant 3, Female PWD, Community focus group 1)

‘Because I am looking after a sick person I can’t even go and look for a job. If I’m not here, it means that he won’t eat the whole day so even if someone asks me to fetch water for R20, I cannot do so.’ (Participant 8, Female Caregiver of a person with a Physical disability, Community focus group 2)

A particular case study discussed in a report on the rights of PWDs from the uMgungundlovu disability forum KwaZulu-Natal DoH (2010) reveals a harrowing tale of a young 15-year-old girl with cerebral palsy. The girl was admitted to a Durban hospital while in labour on the insistence of her neighbour. Social work investigation revealed that this girl was neglected, was locked in a room daily and sexually abused (she was pregnant) and both her parents were alcoholics. Of concern is that she had not been reported by the admitting hospital, despite the obvious signs of neglect and abuse a severely disabled underage girl admitted in a pregnant state in such a severe state of neglect (hair uncared for, tattered clothing, dirty and severe body odour).

While this may reflect on an isolated extreme case, it suggests that some health professionals lacked the ability to recognise signs of neglect and the knowledge on the procedures to follow in the management of such a situation. While this was a blatant case of neglect that one would expect any caring person to respond to, it raises concerns about the ability of health professionals to respond to the more subtle signs of neglect as well as their ability to advocate for their patients without adequate training.

It is imperative that OTs be conscientised; to identify signs of abuse, neglect and marginalisation; and become familiar with processes to ensure that such persons are appropriately and correctly referred and removed from such abusive situations.

From an occupational justice perspective, abuse, marginalisation and isolation of a PWD compromises their safety and security, self-respect, dignity and confidence (Wilcock & Hocking 2015). As such, it leads to greater marginalisation and isolation and seriously impacts on the mental health of the individual concerned. This in turn may compromise self-care and the motivation to engage in essential daily occupations or to seek employment.

Poverty is the underlying cause of these problems. Poverty viewed from a capability perspective maintains that the defining feature of someone who is poor is that they have restricted and limited opportunities to pursue their well-being (United Nations 2004). Poverty results in low levels of capability or, as Sen (1992:107) says, ‘the failure of basic capabilities to reach certain minimally acceptable levels’, which automatically restricts occupational potential. Kronenburg and Pollard (2005) suggest that OTs need to negotiate with communities to obtain their consensus before such occupational injustices can be rectified and social inclusion of PWDs can be facilitated. This would also entail negotiation with governmental and non-governmental organisations to ensure stakeholder buy-in in the community.

The second core theme that emerged from the data was related to OT opportunities for intervention

The first opportunity for intervention emerged from participants’ need for adequate and more appropriate rehabilitation services. PWDs expressed a strong need to access more appropriate assistive devices such as wheelchairs more suited for use in the rural terrain. PWDs also expressed a need for training of their caregivers in issues such as how to transfer the person from floor to wheelchair and back to improve the PWDs ability to move around their home rather than being stuck on the floor. Participants thought it beneficial to have access to an OT more regularly at a PHC level. The PWDs thought it ideal if the OT could do home visits, which would improve OTs insight into the specific challenges facing the PWD as well as inform more contextually relevant application of intervention. Visagie, Scheffler and Schneider (2013) identified gaps in the implementation of wheelchair policy and stated that the current application had a negative impact on users and service providers. WHO (2011) reiterates it is the responsibility of the healthcare system to ensure that there are effective measures in place to promote personal mobility for persons with disabilities, and to ensure availability and accessibility of appropriate assistive devices.

In this regard, the authors agree that OT training be extended to improve the ability to prescribe appropriate assistive devices and that OTs ensure that caregivers and PWDs are taught about the use and care of their assistive devices.

PWDs said that they required assistance to acquire alternative methods to complete basic daily tasks. At the time of the study, some PWDs had seen an OT once at the hospital or clinic but were not currently receiving any therapeutic interventions. The following quotes reveal some of their concerns:

‘I cannot bath myself properly but I try, when there is no-one in the house I have to cover myself with a blanket because I can’t dress myself.’ (Participant 5, Female PWD, Community focus group 1)

‘He [*PWD*] needs to be shown how to crawl into the wheelchair so that when mother is gone to work, he can get some air.’ (Participant 24, Female caregiver of a person with a physical disability, Community focus group 5)

‘The Department of Health could organise visits in the community with disabled people, to do regular check-ups and help us exercise because we spend so much time sleeping, maybe the therapists’ can help.’ (Participant 22, Female PWD, Community focus group 4)

‘They [*OT*] can help because there are different ways of doing things. If they [*OT*] can tell us this, maybe we can get help. (Participant 10, Female PWD, Community focus group 2)

‘One patient who was disabled with his hands he couldn’t dress himself, couldn’t even tie his shoe laces.’ (Participant 29, Female CHW, semi-structured interview 1)

**FIGURE 4:**
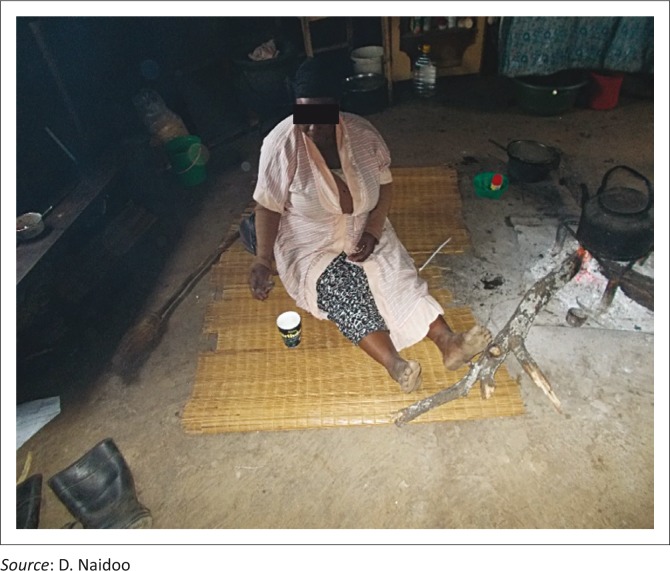
This lady who had stroke battles to cook while on the floor in a rondawel.

**FIGURE 5:**
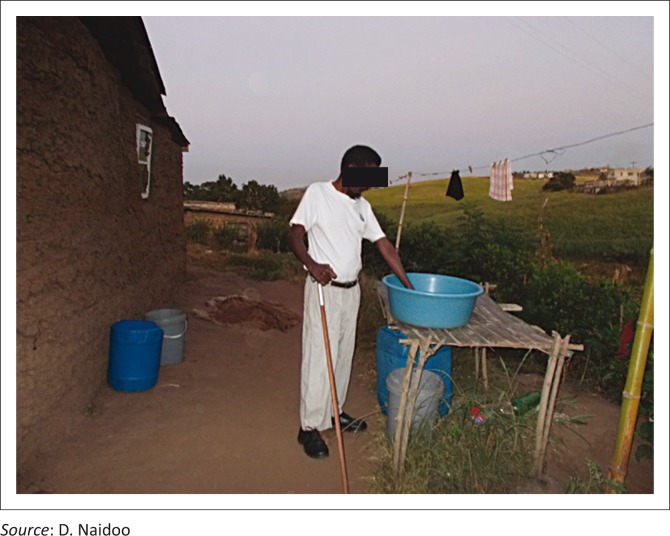
A physically disabled man finding adapted methods to complete his laundry.

Figures 4 and 5 illustrate some of the challenges faced by PWD in performing their daily tasks.

Concurrent with the concerns raised above was a need for greater intervention and assistance relating to the area of mental health, particularly for those people struggling with substance abuse. The need for education around mental health disorders was expressed by CHWs. The following comments illustrate these concerns:

‘He [*mentally disabled person*] needs that injection because he becomes normal with it and we become a family. The kids from our neighbours are scared to walk past our home because he calls them ‘tokiloshe’ [*mythical evil character*].’ (Participant 2, Female caregiver of a person with a mental health disorder, Community focus group 1)

‘It’s just that there isn’t enough assistance for them; some of my clients haven’t been assisted because of my lack of education about mental health.’ (Participant 29, Female CHW, semi-structured interview 1)

A second opportunity for OT intervention that emerged from this theme was the need for support groups to educate about prevention, health promotion and income generation.

As touched on in core theme 1, the lack of awareness of their rights and interventions to enhance their quality of life was evident in the feedback shared by the stakeholders. PWDs thought support and/or counselling groups would provide opportunities for themselves and their caregivers to share stories, gain emotional support and learn from their peers. They believed that education groups with members of the local community could reduce the stigma about disability. They also requested skills training to assist them in income generation to contribute towards the financial needs of their families and households and to boost their disability grant. The following dialogues provide examples:

‘Those who are living with the disabled should receive regular counselling because it is not easy for them to look after a sick person.’ (Participant 7, Female caregiver of a person with a physical disability, Community focus group 2)

‘In other places, disabled people are taken care of because skilled people will come and teach them. The government helps them to use their skills and add to the grant they receive. There is no programme like that here.’ (Participant 13, Male PWD, Community focus group 3)

‘Some of those physically disabled can still think positive things. I remember one person who had an idea of making cupboards he met up with other people but he did not know how to do business plan or how to get money to start.’ (Participant 12, Female CHW, CHW focus group 1)

Health prevention and promotion are key pillars in the PHC approach and feature as the central rationale for OTs to establish support groups with PWDs and their caregivers as well as conducting in-service training for CHWs to broaden their skills and knowledge base (Dawad & Jobson, 2011). The groups and the in-service training would provide a secondary prevention service aimed at creating an opportunity for PWDs and their caregivers to socialise and gain emotional support through sharing their stories, learning from each other and being aware of their rights, which would build capacity, encourage participation and change PWDs’ attitudes as supported in the research by Rule (2013) and Sherry (2014). Peer support groups would further assist in improving assertiveness and activism as peer support from people with similar impairments were found to reduce negative thoughts, lack of expectations and feelings of helplessness (Visagie & Swartz 2016).

OTs involvement in the community would assist with identifying community-specific determinants of the burden of disease. OTs need to partner with the community to develop and implement health promotion programmes to ensure sustainable culturally relevant intervention that addresses the identified problems or health behaviours (Visagie & Schneider 2014).

From an occupational justice perspective, collocating with the community and PWDs would create a better foundation for rights-driven advocacy initiatives to enhance resources, facilitate social inclusion in the community and create opportunities for PWDs to improve their capacity to engage in corporate income generation initiatives. These findings concur with community development principles (Rule et al., 2004; Lorenzo 2003). OT graduates need to acquire skills in empowerment, negotiation, networking, community management, advocacy, intersectoral collaboration, identifying key people in the community who can influence decision-making positively thereby ensuring they are doing with and not doing for PWDs (Lorenzo 2003). This research suggests that there is a need for a shift towards more indirect occupational therapy and less direct (hands on) OT services in order for OTs to have a positive impact on health and well-being outcomes for PWDs.

A third opportunity for intervention was for children with disabilities.

Despite a policy for inclusive education and training of children with disabilities in place, the implementation of this policy at the grassroots level is problematic. This has led to the Human Rights Watch assertion that ‘South Africa has failed to guarantee the right to education for many of the country’s children and young adults due to widespread discrimination against children with disabilities in enrollment decisions’ (Human Rights Watch 2015:1). These findings were confirmed by our study. Mothers of children with disabilities requested assistance with accessing schools for their children. They required information on admission and assessment procedures. Children with physical disabilities were often denied access to mainstream schools because of stereotypical beliefs of the educators.

Additionally, mothers of children with disability expressed disillusionment at the lack of visible progress because of intervention. Mothers were disillusioned at having spent money to access rehabilitation but that these investments did not make a difference in the level of their child’s functioning. The following statement acts as an example:

‘I think that if he can find a school it would be better because I can’t teach him most of the things that are taught in the disabled schools.’ (Participant 33, Female caregiver of a child with a physical disability, Community semi-structured interview 5)

OTs as advocates need to speak up and negotiate for children with disabilities and their rights to be included in school. Therapists need to collaborate with appropriate governmental and social welfare agencies to access the system available for children with disabilities. Therapists could advocate with the department of education to ensure that children with disabilities are placed preferably in mainstream schools to promote their inclusion. Therapists’ could provide a secondary prevention service through providing education to teachers for early identification of children with learning disabilities’ in schools and to facilitate better classroom accommodation of these children’s various disabilities.

The Human Rights Watch further found that:

A lack of understanding of children’s disabilities and a lack of adequate teacher training means that many teachers and school officials do not know how to work with children with disabilities in classrooms. … In some cases, children suffered physical violence and neglect in schools. (Human Rights Watch 2015:2)

This substantiates the need for CHW, caregiver and teacher training that was expressed in this study.

From an occupational justice perspective, the obviously poor prospects for education for children with disabilities in these communities create a foundation for injustice, which thrives in the absence of educational grounding and social awareness. The exclusion of children with disabilities from schools and community activities further marginalises this vulnerable group and excludes them from being involved in income-generating activities thus fostering a lifetime of dependence and isolation

## Conclusion

This study was conducted to understand the particular context in which PWDs lived in semi-rural and rural areas of KZN, the daily challenges they experience and to explore their perceptions of the services required from occupational therapists. The findings reveal the often severe degree to which some PWD and their caregivers live in unsafe and disabling environments preventing them from accessing their equal rights to participating in their daily activities such as self-care, work and social activities. Findings suggest that therapists, health organisations, community stakeholders and professional bodies need to advocate for human resources to ensure sufficient posts for therapists and CHWs to ensure effective community rehabilitation at the PHC level. Much has been done around writing policies; however, implementing them to ensure more accessible and efficient rehabilitation and health promotion and prevention services may remain a utopian dream unless the government implements a long-term commitment to ensure a more effective system of procurement of assistive devices and removal of bottlenecks in services delivery, such as adequacy of posts for therapists at a PHC level to enable therapists working in the community to realise their specific role here. Logistic issues such as providing transport for OTs to travel between home visits and clinics also needs to be addressed as well as review of policy regarding support for disabled to afford to access transport.

OTs need to become serious advocates for PWDs around issues such as access to assistive devices and equal rights, especially as this relates to occupational justice. Therapists also need to take into account the particular uniqueness of the rural community context in SA when facilitating/modifying the environment for the PWD, negotiating ideas to allow for better engagement with daily life tasks and methods to reduce marginalisation and promote both their dignity and greater inclusion of PWDs in the their community.

Higher education programmes have to revise their curricula to ensure that OT graduates have the knowledge and skills necessary to achieve these ends. Health education programmes should be cautious not to fall into a ‘one size fits all’ complaisance in which the emphasis is on a neutralised, generic curriculum that mostly accommodates a middle-class, reasonably resourced, urban-based population’s needs and fails to accommodate the very real and complex differences in needs of PWDs or at risk of disability from an under-resourced, impoverished and mostly peri-urban or rural context.
